# Mapping research trends in macrophage polarization and immunotherapeutic potential in prostate cancer: a bibliometric and visual analysis

**DOI:** 10.3389/fonc.2026.1784198

**Published:** 2026-05-04

**Authors:** Pengze Wu, Lin Chen, Jin Yang, Chengwu He, Aifa Tang, Yafei Yang

**Affiliations:** 1 Department of Clinical Medicine, North Sichuan Medical College, Nanchong, Sichuan, China; 2 Department of Urology, Affiliated Hospital of Chengdu University, Chengdu, Sichuan, China; 3 Department of Urology, The Eighth Affiliated Hospital, Sun Yat-sen University, Shenzhen, Guangdong, China; 4 Department of General Practice Medicine, Third Affiliated Hospital of Shenzhen University (Luohu Hospital Group), Shenzhen, Guangdong, China; 5 Department of Urology, The Third Affiliated Hospital of Shenzhen University (Luohu Hospital Group), Shenzhen University, Shenzhen, Guangdong, China

**Keywords:** immunotherapy, macrophage polarization, prostate cancer, tumor microenvironment, tumor-associated macrophages

## Abstract

**Background:**

Macrophage polarization plays a critical role in shaping the immunosuppressive tumor microenvironment (TME) of prostate cancer (PCa). Tumor-associated macrophages (TAMs), particularly those exhibiting immunoregulatory and tumor-promoting transcriptional programs, contribute to disease progression, immune evasion, and therapeutic resistance.

**Objective:**

To comprehensively map the research landscape of macrophage polarization and activation in PCa using bibliometric tools and to assess translational progress through a review of clinical trials.

**Methods:**

A bibliometric analysis was conducted using VOSviewer, CiteSpace, and R, based on publications related to macrophage polarization and activation in PCa. Additionally, eligible interventional clinical trials in prostate cancer were identified through predefined searches of ClinicalTrials.gov and PubMed, and 20 trials were included in the descriptive synthesis.

**Results:**

Bibliometric findings revealed growing research interest in macrophage polarization since 2015, with key themes including immune suppression, cytokine signaling, and therapeutic resistance. High-frequency keywords highlighted macrophage plasticity/heterogeneity (often captured by M1/M2-related terms in the literature), immune checkpoints, and TME reprogramming. Clinical trials investigated a range of strategies, including CSF1R inhibition (e.g., cabiralizumab, PLX3397), TAM reprogramming strategies, checkpoint blockade, and combination therapies with PARP inhibitors, radiotherapy, and vaccines.

**Conclusion:**

The integration of bibliometric insights with clinical trial and published data highlights the increasing translational focus on TAM/TME-targeted therapies in PCa. These findings underscore macrophage polarization as a promising immunotherapeutic axis and emphasize the need for further clinical innovation and biomarker-driven strategies.

## 1 Introduction

Prostate cancer (PCa) is the second most common malignant tumor among men worldwide, accounting for 13.5% of all male cancer cases (1). In 2025, the American Cancer Society estimates approximately 313,780 new cases and 35,770 deaths from PCa (2). Localized prostate cancer is often effectively managed with radical prostatectomy, radiotherapy, or androgen deprivation therapy (ADT). However, a substantial proportion of patients eventually develop castration-resistant prostate cancer (CRPC), for which therapeutic options remain limited and often palliative. Although ICIs have achieved breakthroughs in various solid tumors, their response rate in prostate cancer remains less than 5%, primarily due to the highly immunosuppressive tumor microenvironment (TME) (3, 4). Increasing attention has been directed toward the role of the tumor microenvironment (TME) in driving PCa progression, immune evasion, and resistance to therapy.

TME refers to the tumor cells and their surrounding milieu, primarily composed of immune and inflammatory cells, cancer-associated fibroblasts (CAFs), adjacent stromal tissue, tumor vasculature, and various cytokines and chemokines (5, 6). Unlike the environment of normal cells, the TME exhibits distinct characteristics that support tumor cell proliferation and survival (7). A hallmark of prostate cancer is the exhaustion of anti-tumor T lymphocytes coupled with abnormal infiltration of pro-tumorigenic macrophages (8). Macrophages constitute approximately 30–50% of immune infiltrates within the tumor microenvironment (TME) and originate from both embryonic and bone marrow–derived developmental pathways. These cells exhibit remarkable plasticity and form a highly heterogeneous population. Historically, macrophage activation has been described using the M1/M2 polarization framework derived from *in vitro* stimulation models, in which macrophages are polarized by defined stimuli such as LPS/IFN-γ or IL-4/IL-13. However, macrophages in tissues, particularly tumor-associated macrophages (TAMs), rarely conform to these binary states (9). Accordingly, in the present study, M1/M2-related terms are interpreted primarily as historically prevalent descriptors in the literature rather than as fixed biological categories, and greater emphasis is placed on TAM plasticity, heterogeneity, and context-dependent functional states. Instead, they exist along a dynamic continuum of activation states characterized by diverse transcriptional programs, molecular markers, and cytokine profiles. Functionally, TAM subsets may exhibit pro-inflammatory and anti-tumor activities or, conversely, immunoregulatory and tumor-promoting properties depending on the signals present in the microenvironment. These functional states are often associated with distinct metabolic tendencies, such as glycolytic metabolism or oxidative phosphorylation, although these features vary across different TAM populations and tumor contexts (10, 11). Recent studies highlight the regulatory role of macrophage polarization in prostate cancer progression. Yu-Fei Liu et al. (12) reported that gut microbiota-derived short-chain fatty acids (SCFAs) promote prostate cancer progression by inducing tumor cell autophagy and shifting macrophage functional programs toward immunoregulatory, tumor-promoting states, which are often described using “M2-like” terminology in the source literature. Conversely, Lian-Sheng Zhang et al. (13) demonstrated that exosomal miRNA-203 promotes pro-inflammatory, anti-tumor macrophage-associated programs, often labeled as “M1-like” in earlier studies. Although research on macrophage polarization in prostate cancer has yielded promising findings, the mechanisms underlying clinical benefits require rigorous validation. Current investigations focus on both basic science and preliminary clinical studies in this field. However, a comprehensive bibliometric analysis and a detailed synthesis of existing discoveries regarding macrophage polarization remain to be conducted.

In this study, we first conducted a comprehensive bibliometric analysis of global literature on macrophage polarization and activation in prostate cancer from 2005 to 2024. Using VOSviewer, CiteSpace, and R-based bibliometric tools, we analyzed publication trends, high-impact keywords, country and institutional collaborations, co-citation networks, and thematic clusters. To enhance the translational relevance of our findings, we further reviewed 20 eligible interventional clinical trials targeting macrophage-related pathways or broader tumor microenvironment-directed strategies in PCa. These trials were identified through predefined searches of ClinicalTrials.gov and PubMed and screened according to prespecified eligibility criteria. The included trials span various therapeutic modalities, including CSF1R inhibition, checkpoint blockade, TAM reprogramming, and combination strategies involving agents such as PARP inhibitors, vaccines, and stereotactic radiotherapy. Some trials focus on macrophage depletion, while others seek to modulate TAM function or enhance antigen presentation, offering a wide-ranging view of therapeutic innovation in the field.

Together, our bibliometric mapping and clinical trial synthesis provide a comprehensive, integrated view of the research landscape surrounding macrophage polarization in prostate cancer. By bridging foundational research with ongoing translational efforts, this study identifies key research hotspots, collaborative hubs, and promising therapeutic directions. These insights may guide future investigations and inform the development of biomarker-driven, macrophage-targeted therapies that could improve outcomes for patients with advanced prostate cancer. Compared with previous bibliometric analyses in related oncology fields, the present study provides a longer analytical period (2005–2024) and integrates clinical trial evidence targeting tumor-associated macrophages. By combining multiple bibliometric tools (VOSviewer, CiteSpace, and Bibliometrix) with geographical collaboration analysis, this study offers a more comprehensive view of the research landscape and its translational implications.

## 2 Materials and methods

### 2.1 Data source and literature search strategy

The bibliometric data used in this study were retrieved from the Web of Science Core Collection (WoSCC) database (https://www.webofscience.com/), which was used as the primary data source for the bibliometric analysis. WoSCC was selected because it provides standardized full records and cited-reference metadata suitable for co-citation, collaboration, and keyword-based bibliometric analyses. We performed a bibliometric analysis of publications pertaining to macrophage polarization in prostate cancer indexed in WoSCC. Following a comprehensive and systematic literature search, we collected relevant articles and reviews published between January 1, 2005, and December 31, 2024. The search strategy was developed with reference to existing reviews on macrophage polarization in prostate cancer and in consultation with field experts. Literature screening and data extraction were performed independently by two researchers. Any discrepancies were resolved through discussion, and when necessary, a third senior investigator was consulted to reach a consensus. All authors agreed upon the following query: (TS=((macrophage polarization) OR (Activation, Macrophage) OR (Activations, Macrophage) OR (Macrophage Activations))) AND TS=((“prostate cancer” OR “prostatic carcinoma” OR “prostatic neoplasm” OR “prostatic cancer” OR “cancer, prostatic” OR “cancers, prostatic”)). Only documents classified as “articles” or “reviews” and published in English within the specified time frame were included. The search terms were developed based on commonly used terminology in the field and were informed by previously published reviews and highly cited studies on macrophage polarization and tumor-associated macrophages in prostate cancer. Key information including sources, titles, keywords, and reference lists of the exported records was extracted into tab-delimited plain text files to ensure consistency and facilitate further analysis.

Inclusion and exclusion criteria.

To ensure methodological transparency and reproducibility, explicit inclusion and exclusion criteria were applied during the literature screening process. Studies were included if they met the following criteria: (1) publications indexed in the Web of Science Core Collection (WoSCC); (2) articles or reviews focusing on macrophage polarization or macrophage activation in prostate cancer; (3) publications written in English; and (4) studies published between January 1, 2005 and December 31, 2024.

Publications were excluded if they met any of the following criteria: (1) document types other than articles or reviews (such as editorials, meeting abstracts, letters, corrections, or news items); (2) studies unrelated to prostate cancer or macrophage polarization; (3) duplicate records retrieved during the database search process; or (4) publications lacking sufficient bibliographic information for bibliometric analysis. For the clinical trial component, studies were included if they met the following criteria (1): registered interventional studies in prostate cancer (2); investigations related to macrophage-targeted, myeloid-modulating, immunotherapeutic, or broader tumor microenvironment-directed strategies; and (3) availability of sufficient registry or publication information for descriptive analysis. Studies were excluded if they were non-interventional, unrelated to prostate cancer, duplicate records, or lacked adequate information for data extraction.

To complement the bibliometric assessment, we also systematically searched ClinicalTrials.gov and PubMed to identify eligible interventional clinical trials investigating macrophage-related, immunotherapeutic, or broader tumor microenvironment-targeted strategies in prostate cancer. Two investigators independently screened all retrieved records according to prespecified eligibility criteria, and any disagreements were resolved by discussion and consensus. Using this predefined screening process, 20 eligible interventional studies, including randomized controlled trials and early-phase exploratory trials, were ultimately included in **Table 1** to provide clinical context for emerging translational research directions.

**Table 1:** Representative clinical trials of immunotherapy and tumor microenvironment (TME)/tumor-associated macrophage (TAM) modulation in prostate cancer.

NO	NCT number	Trial name/intervention	Study type	Patient population	Therapeutic modality	Status
1	NCT03834506	KEYNOTE-921: Pembrolizumab+ Docetaxel Vs Placebo+ Docetaxel	Phase III RCT	mCRPC	PD-1 inhibitor + chemotherapy	Active, not recruiting
2	NCT02861573	KEYNOTE-199: Pembrolizumab monotherapy	Phase II		PD-1 inhibitor	Completed
3	NCT03338790	CheckMate 650: Nivolumab + Ipilimumab	Phase II		PD-1 + CTLA-4 blockade	Completed
4	NCT02489357	COSMIC-021: Cabozantinib +Atezolizumab	Phase I/II		VEGFR/MET inhibitor + PD-L1 inhibitor	Active
5	NCT04455750	TALAPRO-2: Talazoparib + Enzalutamide	Phase III RCT		PARP inhibitor + AR inhibitor	Active
6	NCT03748641	MAGNITUDE: Niraparib + Abiraterone + Prednisone	Phase III RCT		PARP inhibitor + AR inhibitor	Completed
7	NCT03834519	KEYLYNK-010: Pembrolizumab + Olaparib vs. Abiraterone/Enzalutamide	Phase III RCT		PD-1 inhibitor + PARPi	Terminated
8	NCT03572478	NCT03572478: Sipuleucel-T + Pembrolizumab	Phase II		Cancer vaccine + PD-1 inhibitor	Active
9	NCT00696969	PROSTVAC-VF + GM-CSF	Phase II		Viral vector vaccine	Completed
10	NCT03250057	NCT03250057: Durvalumab + Olaparib	Phase II		PD-L1 inhibitor + PARPi	Completed
11	NCT05075577	NCT05075577: PSMAxCD3 Bispecific Antibody	Phase I		T-cell engager (immunotherapy)	Recruiting
12	NCT03493945	NCT03493945: CD40 agonist + Pembrolizumab	Phase I	Solid tumors incl. PCa	TAM activation + checkpoint blockade	Active
13	NCT03177460	NCT03177460: Cabiralizumab (CSF1R inhibitor) + Nivolumab	Phase I/II		TAM depletion (CSF1R) + PD-1 blockade	Terminated
14	NCT04256307	NCT04256307: PLX3397 (CSF1R inhibitor) + Pembrolizumab	Phase I/II		TAM/TME modulation	Active
15	NCT02499835	ECHO-202: Epacadostat (IDO1 inhibitor) + Pembrolizumab	Phase I/II		TME immunometabolism + PD-1	Completed
16	NCT04109729	NCT04109729: Anti-CD47 antibody (Magrolimab)	Phase I		Macrophage checkpoint blockade (CD47-SIRPα)	Active
17	NCT02985957	JAVELIN Solid Tumor: Avelumab monotherapy	Phase I/II	Advanced PCa	PD-L1 inhibitor	Completed
18	NCT04821622	NCT04821622: PSMA-CAR-T cells	Phase I		CAR-T immunotherapy	Recruiting
19	NCT04336943	NCT04336943: Entinostat (HDAC inhibitor) + Atezolizumab	Phase I/II		Epigenetic + PD-L1 blockade (TME reprogramming)	Active
20	NCT03629756	NCT03629756: PD-1 antibody + SBRT	Phase II	Oligometastatic PCa	Immunotherapy + radiotherapy	Active

### 2.2 Data analysis and graph acquisition

This study employs an integrated multidimensional bibliometric approach to systematically examine research trends in macrophage polarization within prostate cancer. Using WoSCC as our data source, we constructed a specialized literature dataset that was processed and analyzed through the bibliometrix package (version 4.1.4) in R (version 4.3.1), generating a standardized matrix encompassing key metadata elements including authorship, institutional affiliations, country origins, and keyword terms. Our analytical framework combined the complementary strengths of VOSviewer (version 1.6.20) and CiteSpace (version 6.2.R4) to establish a comprehensive network architecture, featuring geographic collaboration patterns visualized through ArcMap 10.8, keyword co-occurrence networks with appropriate frequency thresholds, and temporal citation evolution patterns. The CiteSpace component enabled identification of influential publications and emerging research fronts through its burst detection algorithm (γ=0.5). By integrating VOSviewer’s clustering capabilities with CiteSpace’s log-likelihood ratio analysis, we achieved robust validation of research hotspots. This multidimensional approach yielded a sophisticated analytical matrix that simultaneously tracks temporal progression, geographic distribution, and thematic evolution, thereby providing systematic insights into the field’s knowledge structure dynamics and patterns of international scholarly collaboration.

## 3 Results

### 3.1 Publication overview and workflow

**Figure 1:**
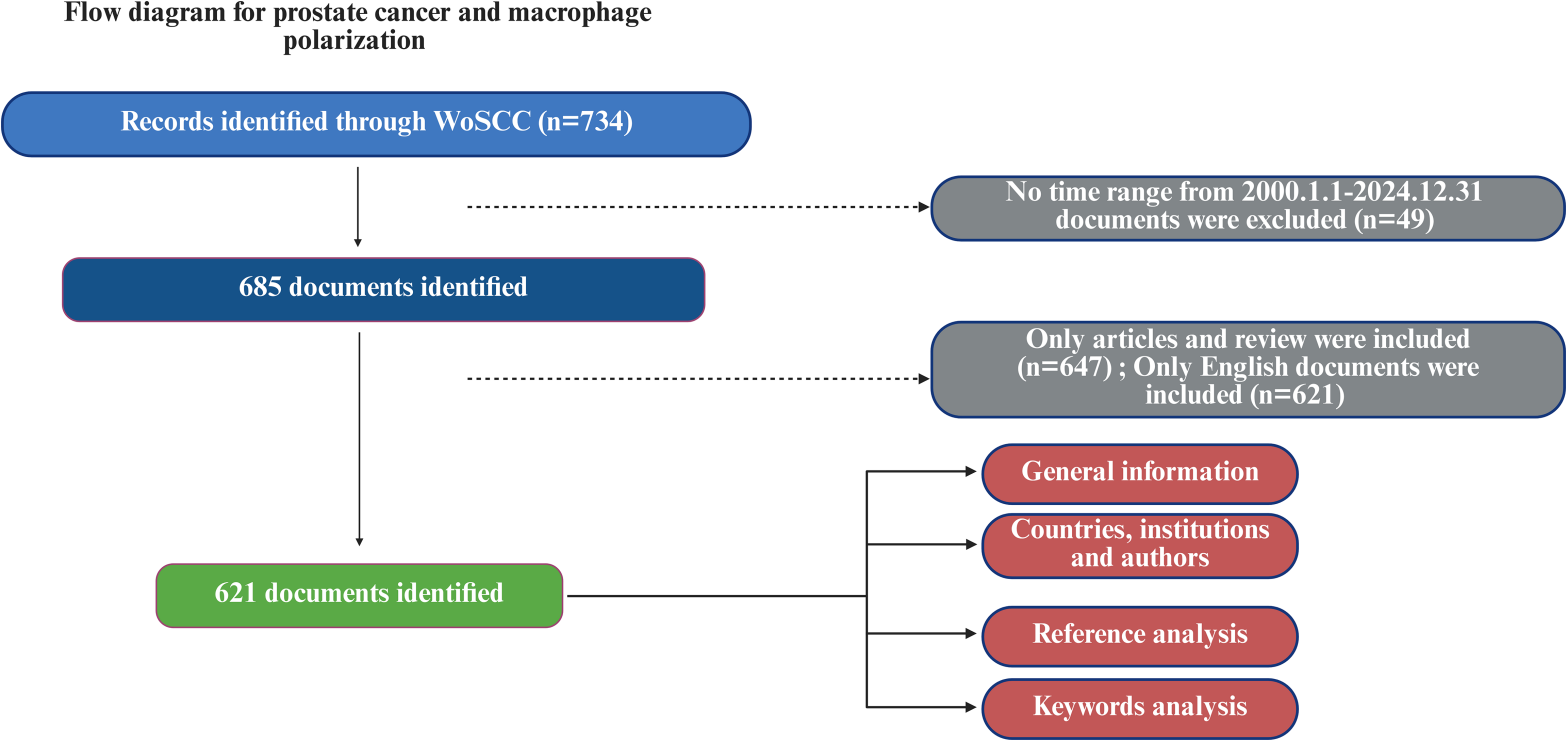
Flow diagram illustrating the literature search, screening, eligibility assessment, and inclusion process for the bibliometric analysis of macrophage polarization and immunotherapeutic potential in prostate cancer. Figure created with BioRender (biorender.com).

As outlined in **Figure 1**, we conducted a systematic bibliometric analysis of macrophage polarization in prostate cancer following a standardized workflow. From an initial retrieval of 734 records in WoSCC, we implemented sequential filters: temporal restriction (2005.01.01-2024.12.31; 685 publications), document-type selection (articles/reviews; 647 publications), and English-language limitation (final 621 publications). **Table 2** reveals an annual growth rate of 5.78% in this research domain during 2005-2024, with the 621 publications collectively cited 34,689 times. Comprehensive bibliometric indicators are presented in our statistical analysis.

**Table 2:** The main information of the data.

Description	Results
Documents	621
Article	511
Review	110
Time span	2005:2024
Sources (journals, books, etc.)	300
Annual growth rate (%)	5.78%
Document average age	8.19
References	34689
Average citations per doc	54.63

### 3.2 Temporal publication trends

**Figure 2:**
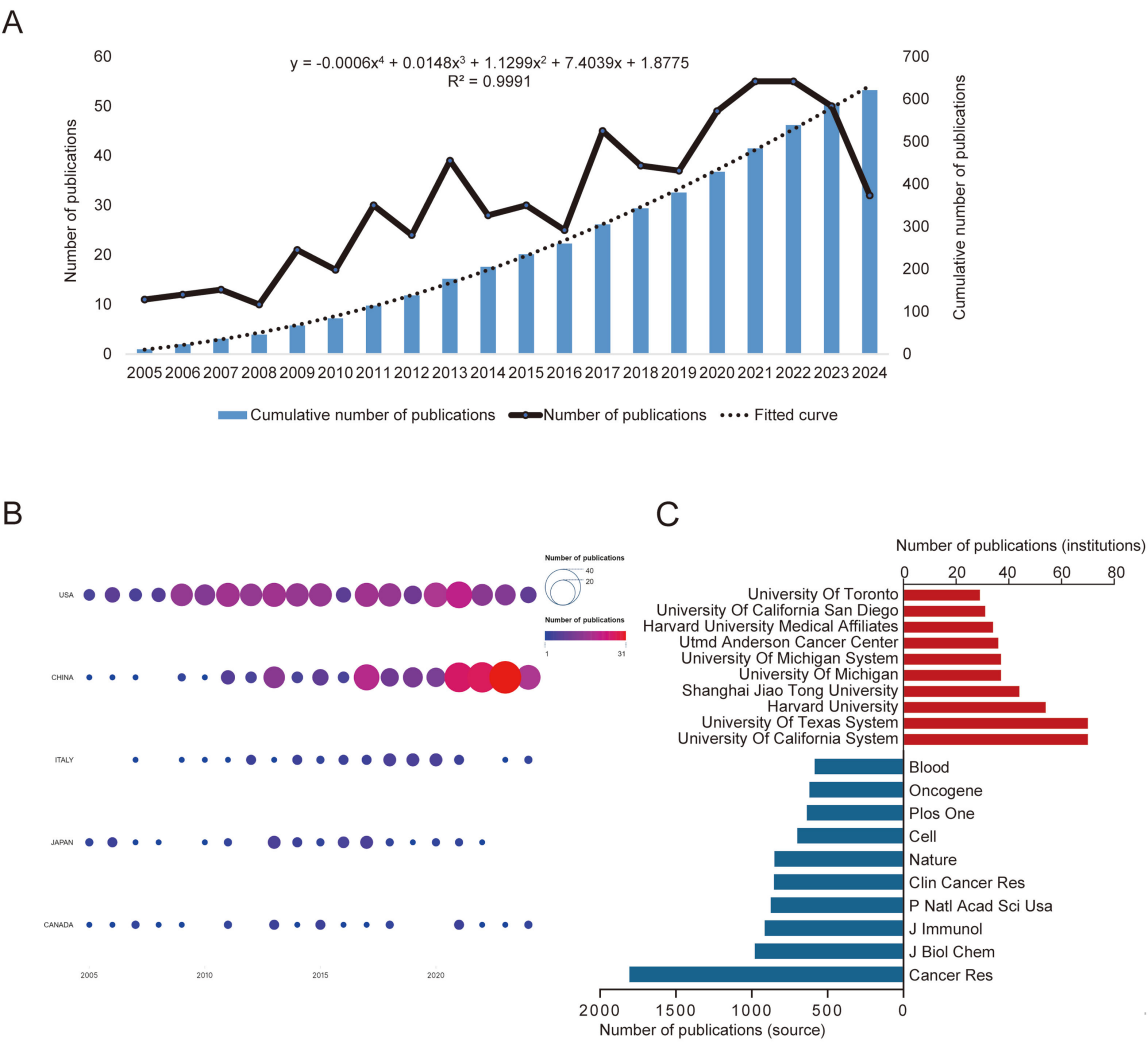
Quantitative analysis of publication status. **(A)** Annual volume of Macrophage polarization in prostate cancer publication from 2005 to 2024. **(B)** Top 10 countries in the field of Macrophage polarization in prostate cancer. **(C)** Top 10 institutions and top 10 journals in macrophage polarization research in prostate cancer (WoSCC, 2005–2024).

To elucidate the evolving research landscape of macrophage polarization in prostate cancer, we systematically analyzed annual and cumulative publication outputs from 2005 to 2024. **Figure 2A** demonstrates sustained growth in annual publications: from merely 11 publications in 2005 to nearly double by 2009, stabilizing at approximately 50 publications annually during 2020-2023. A fourth-order polynomial regression model (y = -0.0006x^4^ + 0.0148x³ + 1.1299x² + 7.4039x + 1.8775; R² = 0.9991) confirmed this significant growth trend. Geospatial analysis of WoSCC data (**Figure 2B**) revealed the United States, China, and Italy as the top three contributing nations, with the US and China demonstrating substantially higher output than other countries (**Supplementary Table 1**). Institutional productivity mirrored national trends: 8 of the top 10 research institutions were American, with the University of California system dominating productivity rankings. The analysis also identified the top 10 contributing journals, led by Cancer Research, Journal of Biological Chemistry, and The Journal of Immunology (**Figure 2C**).

### 3.3 Spatial distribution: countries and institutions

**Figure 3:**
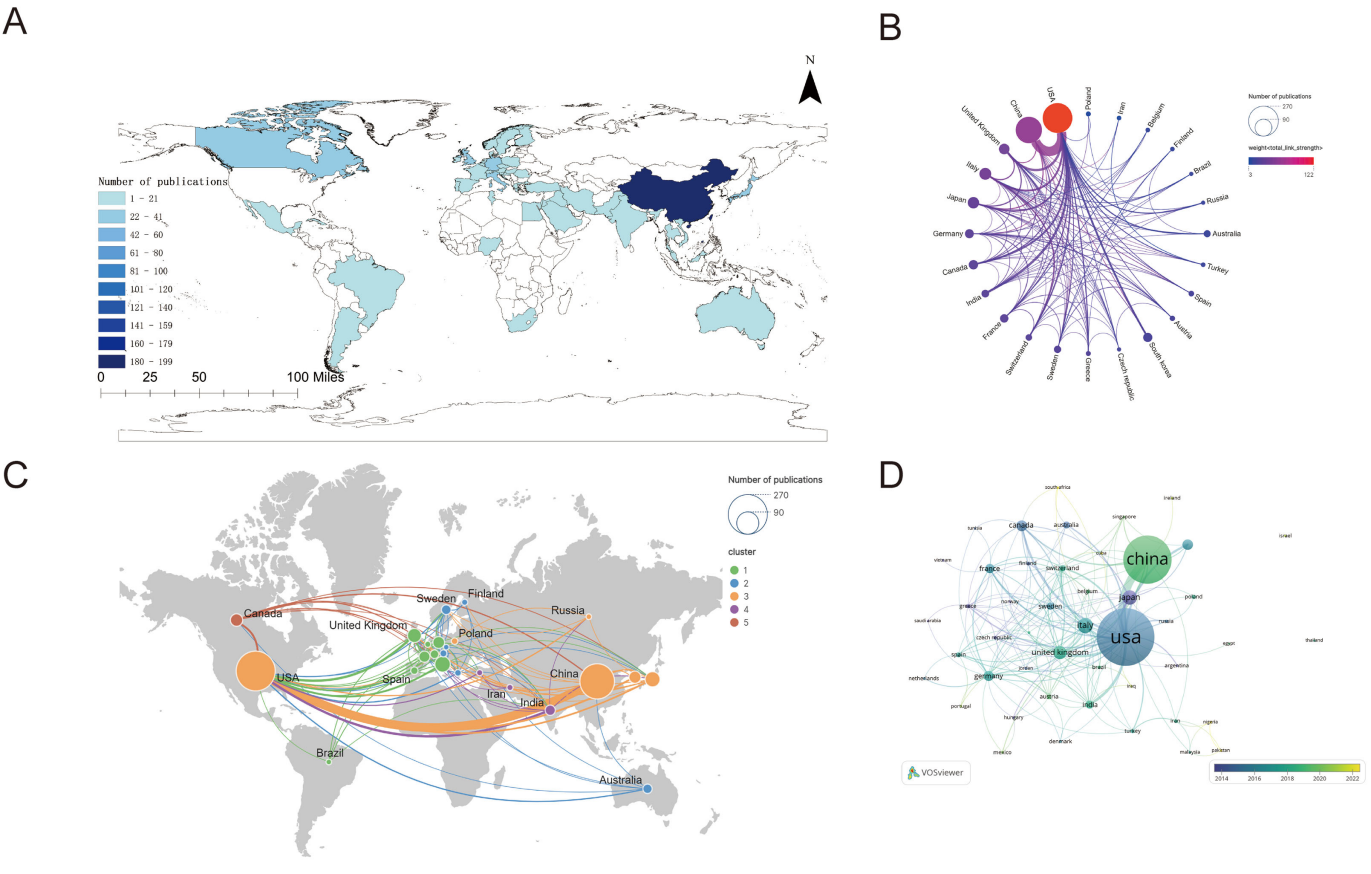
**(A)** Geographic distribution of research contributions from 50 countries investigating macrophage polarization in prostate cancer **(B)** International collaboration network analysis of publication output among contributing nations **(C)** Bibliometric assessment of citation impact versus collaborative engagement across countries **(D)** Temporal evolution of international research partnerships (2005–2024).

Using ArcGIS, we analyzed publication outputs across 51 countries contributing to prostate cancer macrophage polarization research (**Figure 3A**). The United States led with 254 publications, followed by China (199), Italy (40), Japan (37), and the United Kingdom (32), while other countries produced fewer than 30 publications each. Collaboration networks were visualized through VOSviewer and Scimago Graphic, represented by circular diagrams (**Figure 3B**), visualization types (**Supplementary Figure 1**) and world maps (**Figure 3C**). In **Figure 3B**, country-specific circles scaled by publication volume revealed increasing collaboration levels clockwise from Poland to the US. The US emerged as the most active collaborator, with particularly frequent partnerships with China. Other key US collaborators included Russia (9 collaborations), the United Kingdom (9), Canada (7), South Korea (7), and Italy (7). Temporal analysis of national contributions (**Figure 3D**) used color gradients (dark blue to light yellow) to represent publication years. Early research (10+ years ago) was predominantly US-based, while recent studies (past 5 years) showed growing contributions from China and Singapore. This shift may reflect both the rising clinical recognition of macrophage polarization’s role in prostate cancer and the US’s enduring leadership in this field.

### 3.4 Knowledge network: disciplines and co-citation structure

**Figure 4:**
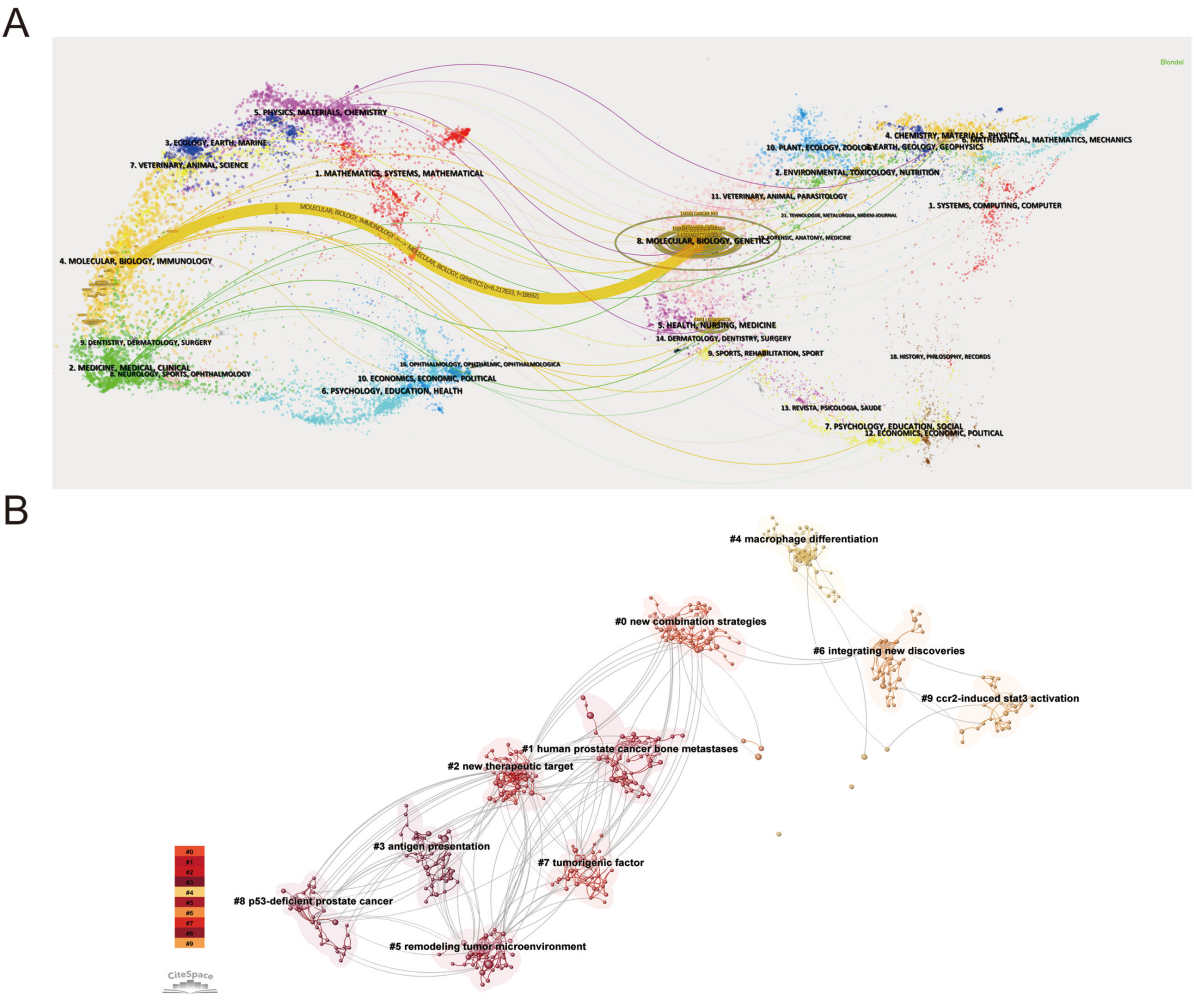
Analysis of the evolution of research disciplines and cocited references **(A)** Dual-map overlay and corresponding disciplines. The citing journals are on the left, the cited journals are on the right, and the colored path represents the citation relationship. **(B)** Cluster analysis of cocited reference networks.

To elucidate citation patterns and thematic distribution across academic journals, we performed a dual-map overlay analysis using CiteSpace (**Figure 4A**). The left-side base map represents citing journals, while the right-side displays cited journals, with each point corresponding to a journal category. The connecting citation curves reveal interdisciplinary linkages, where line thickness indicates citation strength. Our analysis demonstrates that publications on prostate cancer macrophage polarization predominantly cite articles from molecular/biology/genetics journals (strongest connections shown by thickest lines), while being most frequently cited by journals in molecular/biology/immunology domains. These citation trajectories highlight the field’s interdisciplinary nature, particularly the integration of immunological concepts with fundamental cancer biology research.

### 3.5 Analysis of co-cited references and authors

The co-citation network analysis of 34,689 references from 621 publications on prostate cancer macrophage polarization revealed 10 distinct research clusters through CiteSpace’s log-likelihood ratio algorithm (**Figure 4B**). The largest clusters included “Novel combination strategy” (Cluster #0, size=57), “Human prostate cancer bone metastasis” (Cluster #1, size=55), and “New therapeutic target” (Cluster #2, size=50), followed by “Antigen presentation” (Cluster #3, size=45), “Macrophage differentiation” (Cluster #4, size=43), and “Tumor microenvironment remodeling” (Cluster #5, size=40). Smaller but significant clusters comprised “Integrating new discoveries” (Cluster #6, size=40), “Oncogenic factors” (Cluster #7, size=37), “p53-deficient prostate cancer” (Cluster #8, size=37), and “CCR2-induced STAT3 activation” (Cluster #9, size=29). Among these clusters, Cluster #0 (“Novel combination strategy”) represents the dominant translational direction in this field, highlighting combinatorial therapeutic approaches designed to overcome the biological redundancy and adaptive immunosuppressive networks of the prostate tumor microenvironment. Representative studies within this cluster integrate macrophage-targeted or myeloid-modulating strategies with immune checkpoint blockade, targeted therapies, radiotherapy, vaccines, or metabolic interventions. Rather than supporting single-pathway inhibition alone, this cluster suggests that effective treatment may require coordinated reprogramming of multiple components of the tumor microenvironment, including TAM function, antigen presentation, stromal signaling, and immunometabolic interactions. In prostate cancer, such strategies often involve attempts to modify TAM-mediated immune suppression while simultaneously enhancing anti-tumor immune responses. These clusters naturally grouped into three interconnected research themes: the first encompassing pathogenic mechanisms through Clusters #1, #3, #4 and #9 which explore fundamental processes of macrophage polarization; the second represented by Clusters #0, #2, #5 and #6 focusing on translational applications and therapeutic development; and the third comprising Clusters #1 and #8 that highlight disease-specific contexts in prostate cancer subtypes. This comprehensive mapping demonstrates how the field systematically bridges mechanistic understanding of macrophage polarization with clinical applications while addressing pathological diversity in prostate cancer progression.

Meanwhile, **Supplementary Figure 2** displays the top 10 most prolific authors in this field. Notably, Pienta KJ emerged as the most productive author with 10 publications, while Liu Y and Wang J ranked joint second with 8 publications each.

### 3.6 Analysis of top-cited articles

As a core component of bibliometric methodology, citation analysis forms the basis for impact factor evaluation, where highly cited publications are recognized as scientifically significant works that establish research directions in their field. **Table 3** presents the top 10 most cited articles ranked in descending order by citation count (14–23). Notably, the majority of these seminal works were published between 2009 and 2015, collectively accumulating 6,512 citations, with individual citation counts ranging substantially from 437 to 1,144. This concentration of influential publications within a defined timeframe suggests a period of particularly impactful research development in prostate cancer macrophage polarization studies.

**Table 3:** The top 10 papers with the highest number of citations.

Rank	Title	Key points	Journal	Local citation	Year
1	Tumor-associated macrophages (TAM) as major players of the cancer-related inflammation (14)	Summarizes tumor-associated macrophages' roles in promoting cancer progression and highlights their potential as therapeutic targets within the inflammatory tumor microenvironment.	J Leukoc Biol	1144	2009
2	Carcinoma-produced factors activate myeloid cells through TLR2 to stimulate metastasis (15)	Identifies tumor-secreted versican as a TLR2/6 activator in macrophages, promoting TNF-α–driven inflammation and metastatic progression in the tumor microenvironment.	Nature	901	2009
3	Vascular normalizing doses of antiangiogenic treatment reprogram the immunosuppressive tumor microenvironment and enhance immunotherapy (16)	Demonstrates low-dose anti-VEGFR2 therapy normalizes vasculature, repolarizes macrophages to M1 phenotype, and enhances T-cell infiltration and vaccine efficacy in prostate cancer models.	Proc Natl Acad Sci U S A	842	2012
4	Iron oxide nanoparticles: Diagnostic, therapeutic and theranostic applications (17)	Reviews iron oxide nanoparticles for diagnostics and therapy, highlighting their role in macrophage polarization, image-guided treatment, and theranosticapplications in cancer.	Adv Drug Deliv Rev	794	2019
5	lncRNA-dependent mechanisms of androgen-receptor-regulated gene activation programs (18)	Identifies lncRNAs PRNCR1 and PCGEM1 as enhancers of androgen receptor signaling, promoting ligand-independent prostate cancer growth and castration resistance.	Nature	546	2013
6	B-cell-derived lymphotoxin promotes castration-resistant prostate cancer (19)	Links androgen ablation to inflammatory cell infiltration in prostate cancer, where IKKβ/STAT3 activation promotes castration resistance and hormone-independent tumor survival.	Nature	491	2010
7	Metformin inhibits the senescence-associated secretory phenotype by interfering with IKK/NF-B activation (20)	Shows metformin inhibits NF-κB signaling and inflammatory cytokine expression, reducing senescence-associated prostate cancer growth independent of AMPK activation.	Aging Cell	454	2013
8	Safety and Survival With GVAX Pancreas Prime and Listeria Monocytogenes-Expressing Mesothelin (CRS-207) Boost Vaccines for Metastatic Pancreatic Cancer (21)	Demonstrates that GVAX/CRS-207 vaccination enhances mesothelin-specific T-cell responses and significantly improves survival in pancreatic cancer with minimal toxicity.	J Clin Oncol	453	2015
9	CCL2 and Interleukin-6 Promote Survival of Human CD11b+ Peripheral Blood Mononuclear Cells and Induce M2-type Macrophage Polarization (22)	Shows CCL2 and IL-6 promote M2 macrophage polarization in prostate cancer by inhibiting caspase-8 cleavage and enhancing autophagy, supporting tumor progression.	J Biol Chem	450	2009
10	Clinical significance of macrophage heterogeneity in human malignant tumors (23)	Reviews tumor-associated macrophage polarization toward the M2 phenotype, emphasizing their protumoral roles in angiogenesis, immunosuppression, and tumor progression in human cancers.	Cancer Sci	437	2014

### 3.7 Frequency and clustering analysis of keywords

**Figure 5:**
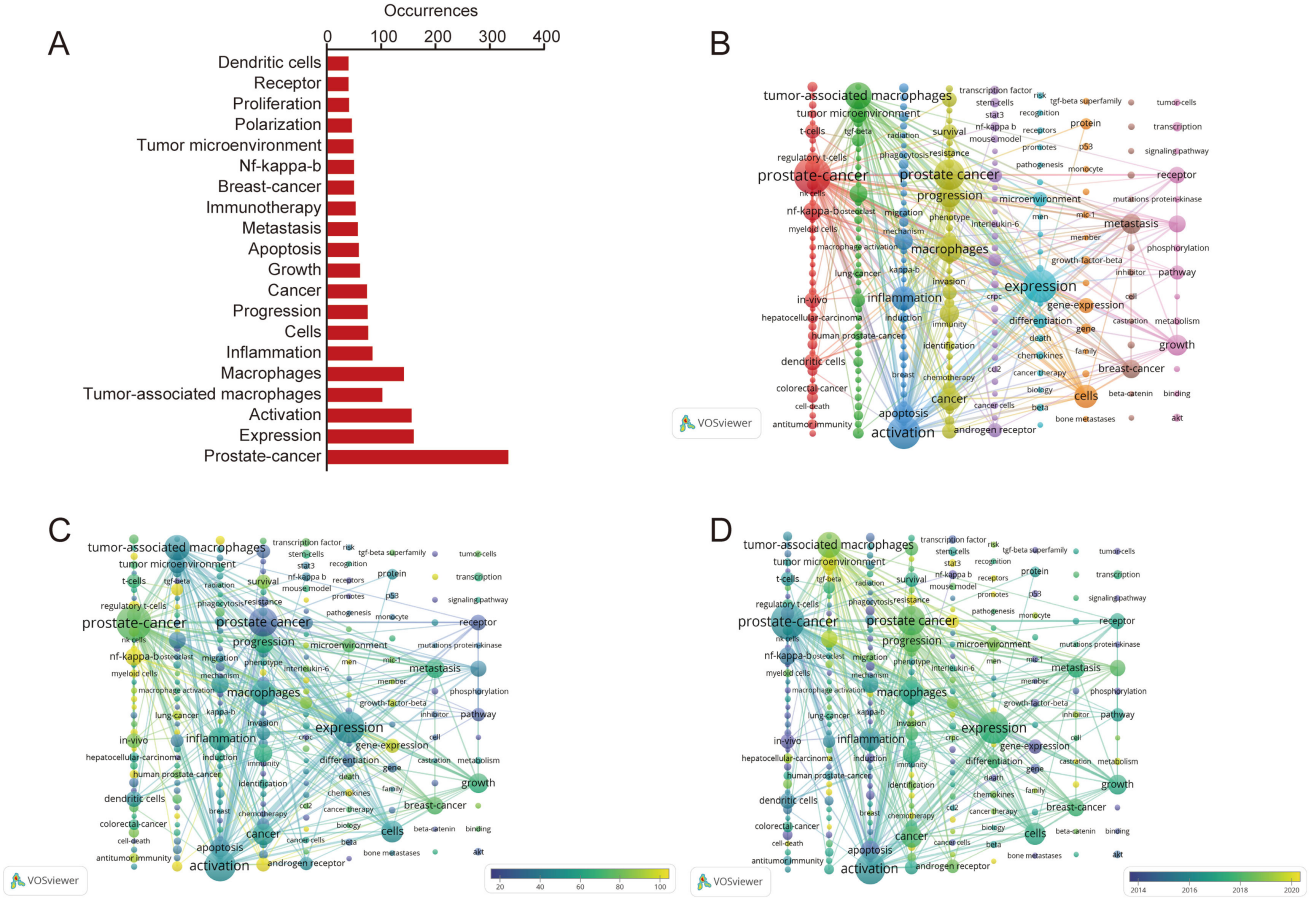
Network of related keywords in the study of macrophage polarization in prostate cancer (2005–2024). **(A)** The top 20 keywords in the field of macrophage polarization in prostate cancer. **(B)** keyword cluster analysis. **(C)** cocited network of macrophage polarization in prostate cancer. **(D)** temporal evolution of the co-occurrence keyword network.

Keywords in scientific publications systematically characterize core research content, and their co-occurrence networks effectively identify research hotspots within a field. In this study, Frequency analysis (**Figure 5A**) revealed “prostate cancer” (N = 334) as the most prevalent keyword, followed by “expression” (N = 160) and “activation” (N = 156). Notably, “breast cancer” (N = 50) emerged as the only malignant tumor subtype ranked among the top 20 keywords (**Supplementary Table 2**). Hierarchical clustering categorized high-frequency keywords into nine distinct thematic clusters (**Figure 5B**). Integrated analysis of co-occurrence network topology (**Figure 5C**) and temporal evolution patterns (**Figure 5D**) demonstrated a significant paradigm shift in research focus: from initial investigations into physiological mechanisms to current emphasis on clinical diagnostic value assessment and therapeutic target identification in prostate cancer.

### 3.8 Further analysis of macrophage polarization in prostate cancer

**Figure 6:**
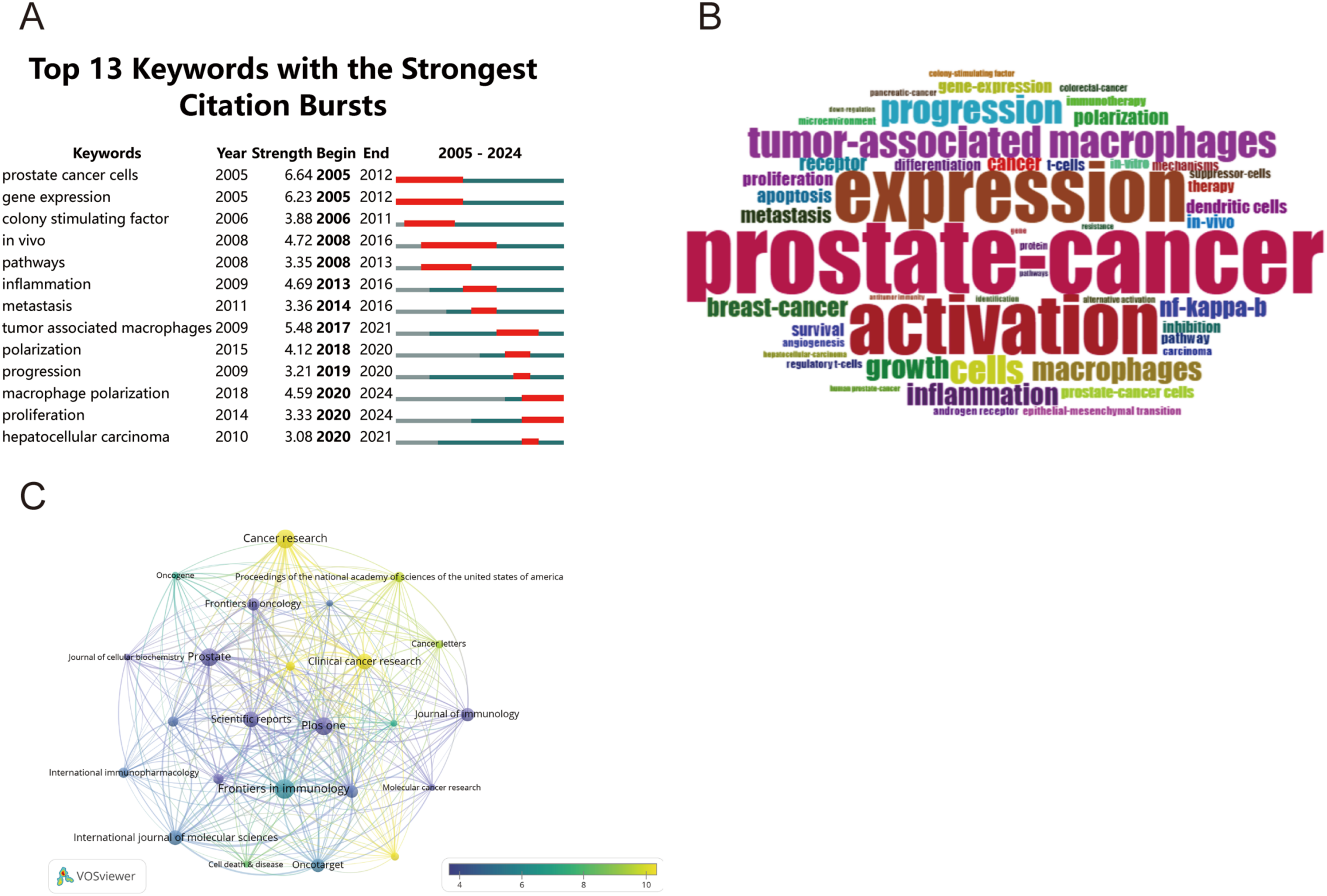
Comprehensive analysis of macrophage polarization in prostate cancer research: **(A)** temporal trends of the top 13 keywords with the strongest citation bursts; **(B)** word cloud visualization of frequently occurring keywords; **(C)** journal network visualization based on bibliometric mapping.

Further analysis of macrophage-related research in prostate cancer showed that the keyword burst analysis (**Figure 6A**) identified 15 terms with sustained citation activity for at least one year. Among these, “*in vivo*” (2008–2016) demonstrated the longest period of attention, whereas more recent bursts included “macrophage polarization” (2020–2024), “proliferation” (2020–2024), and “tumor-associated macrophages” (2017–2021). These findings suggest a growing focus on macrophage functional regulation, immune suppression, and tumor microenvironment remodeling in prostate cancer. The word cloud visualization (**Figure 6B**) likewise indicates sustained interest in TAM-related mechanisms within the prostate TME. However, because keyword-based bibliometric analysis reflects the terminology used in published studies, the resulting atlas is more sensitive to historically dominant descriptors such as “polarization” than to newly emerging macrophage subpopulations defined by single-cell or spatial profiling. Current investigations employing *in vivo* models aim to elucidate how TAMs influence cancer cell proliferation and how inflammatory signaling drives prostate cancer progression, potentially informing novel therapeutic strategies targeting the immune microenvironment. Journal co-citation analysis through VOSviewer (**Figure 6C**) mapped the publication landscape, where circle size corresponds to publication volume and color gradient (blue to yellow) represents journal impact factor. The connecting lines illustrate co-citation relationships among journals, highlighting key knowledge networks in this research domain.

## 4 Discussion

### 4.1 General information of findings

Our analysis of 621 studies on macrophage polarization in prostate cancer published between January 2005 and December 2024 revealed a consistent growth trajectory in annual publication output. A notable surge in publications occurred after 2009, with annual output nearly doubling. This trend may partly stem from the influential study by Solinas et al. (14), which systematically defined the role of tumor-associated macrophages (TAMs) in cancer-related inflammation and highlighted their therapeutic potential within the tumor microenvironment. In addition to this seminal work, the rapid development of tumor immunology during the late 2000s and early 2010s further stimulated research interest in macrophage polarization. Increasing evidence during this period demonstrated that macrophages are key regulators of immune suppression, angiogenesis, and metastatic progression in multiple cancer types. At the same time, technological advances such as high-throughput sequencing and improved immune profiling methods enabled deeper characterization of macrophage heterogeneity within tumors.

From 2019 to 2023, annual publication numbers stabilized at approximately 50 articles. This plateau may indicate that the field has entered a relatively mature phase, in which research is gradually shifting from exploratory identification of macrophage polarization phenomena toward mechanistic studies and translational strategies aimed at modulating tumor-associated macrophages in cancer therapy. The sustained productivity reflects both the fundamental importance of macrophage polarization mechanisms in prostate cancer biology and their translational relevance for therapeutic development.

### 4.2 Leading countries and institutions

Analysis of prostate cancer macrophage polarization research from 2005 to 2024 reveals participation from 50 countries worldwide, demonstrating broad international interest in this field. However, research productivity remains concentrated in developed nations, with underdeveloped countries contributing disproportionately less - a disparity potentially attributable to multiple barriers including inadequate funding (leading to equipment shortages and disrupted research continuity), brain drain of skilled researchers, and marginalization from international collaborative networks that perpetuates resource monopolization. Notably, China emerges as an exceptional developing nation, ranking among the top contributors with 199 publications, likely benefiting from its national strategy integrating TME research into precision medicine initiatives. However, despite ranking second in total publication output, the level of international collaboration involving Chinese institutions appears relatively limited compared with that of the United States and several European countries. This pattern may be influenced by differences in research funding structures, national scientific priorities, language barriers, and established collaboration networks. Strengthening international collaborations could further enhance knowledge exchange and accelerate the global development of research on macrophage polarization in prostate cancer. Through specialized talent programs attracting both domestic and international immunology experts, China has significantly strengthened its research capacity in this field. This contrast underscores the pivotal role of national-level research investment and policy direction in overcoming systemic resource barriers and achieving scientific advancement.

Our analysis suggests researchers should particularly focus on Cancer Research, Journal of Biological Chemistry, and The Journal of Immunology (**Figure 2C**), as these journals are frequently cited in the field’s literature. These high-impact publications consistently feature foundational studies and breakthrough advances that have significantly contributed to our understanding of macrophage polarization in prostate cancer. The substantial citation frequency of works published in these journals underscores their central role in disseminating pivotal findings that drive both mechanistic discoveries and clinical translation in this research domain.

### 4.3 Hotspots and trends

Over the past two decades, research on prostate cancer within the context of tumor immunology has undergone a pronounced intellectual and thematic shift, moving from descriptive and phenomenological studies toward mechanistic and therapeutically oriented investigation. This evolution is clearly reflected in keyword burst and co-occurrence patterns. The early strength of terms such as gene expression and colony stimulating factor during the period 2005 to 2012 signifies an initial phase focused on identifying key molecular players and signaling pathways in prostate cancer cells and their inflammatory microenvironment. Subsequently, the rising emphasis on concepts including tumor-associated macrophages, polarization, and metastasis marked a critical transition, recognizing macrophage plasticity as a central regulator of disease progression. The continued prominence of macrophage polarization and proliferation into the present underscores a research paradigm now concentrated on deciphering molecular drivers of pro-tumorigenic polarization, with current studies exploring mechanisms such as endocytosis, TAM receptor signaling involving MerTK and Axl, and epigenetic regulation. The sustained attention to these themes in high-impact journals reflects a bidirectional flow of inquiry where mechanistic insights inform therapeutic applications, and clinical observations stimulate fundamental research. This mature trajectory demonstrates a field increasingly aimed at leveraging mechanistic understanding to develop macrophage reprogramming strategies and overcome treatment resistance in prostate cancer.

### 4.4 Functional heterogeneity and immunomodulatory roles of tumor-associated macrophages in prostate cancer

TME is a highly complex ecosystem comprising cancer cells, stromal components such as cancer-associated fibroblasts and TAMS, along with various non-cellular elements. These components engage in continuous and dynamic interactions (24–26). This system facilitates immune escape and metastasis through multiple mechanisms, beginning with the recruitment of immunosuppressive cells including regulatory T cells and myeloid-derived suppressor cells, and the activation of pro-angiogenic VEGF signaling (27). These processes are precisely coordinated by multi-level signaling networks, particularly the Notch and TGF-β pathways (28–30), and are further reinforced by immune checkpoint molecules such as PD-1 and CTLA-4 (31, 32). Through these mechanisms, various chemokine axes including CCL2 and CCL22 reshape the immunosuppressive microenvironment, establishing an ecological niche favorable for tumor progression. Additionally, exogenous factors such as the gut microbiota introduce further spatiotemporal heterogeneity to this system (33, 34). Metabolic reprogramming processes, including glycolysis and lipid metabolism, not only provide energetic support for tumor proliferation via cancer-associated fibroblasts but also synergize with immunosuppressive cells like TAMS and regulatory T cells to promote chronic inflammation and the formation of pre-metastatic niches, thereby establishing a self-reinforcing vicious cycle (35–37). Current research employs 3D organoid and organ-on-a-chip systems to model the pathophysiological processes of the TME for developing combination therapeutic strategies (38, 39). However, successful clinical translation requires a comprehensive understanding of its spatiotemporal heterogeneity and component interactions.

TAMs, as key regulatory cells within the TME, profoundly influence cancer progression and treatment outcomes due to their remarkable plasticity (40). Originating from peripheral blood monocytes, these cells possess a unique ability to sense and integrate diverse microenvironmental signals, and their functional states exist along a continuum rather than conforming to a simple binary model derived from *in vitro* polarization conditions (41). Although the traditional M1/M2 framework provides a simplified conceptual model, increasing evidence indicates that TAMs *in vivo* represent a spectrum of activation states rather than discrete phenotypes. In this context, binary labels should be interpreted cautiously, particularly in tumor tissues where macrophage states are shaped by spatial location, metabolic constraints, stromal interactions, and treatment exposure. Recent single-cell and spatial profiling studies further suggest that TAM populations may be better described as functionally distinct but overlapping programs, including immunoregulatory, inflammatory, angiogenic, and matrix-remodeling states, rather than as fixed M1 or M2 categories. Many TAM populations display immunoregulatory and tumor-promoting transcriptional signatures that correlate with poor prognosis in solid tumors through mechanisms such as suppression of anti-tumor immunity, promotion of angiogenesis, and enhancement of therapeutic resistance (42). Their polarization is dynamically regulated by tumor-derived factors and exogenous signals, positioning these cells as critical intermediaries linking innate and adaptive immunity (43). Clinically, the TAM-mediated immunosuppressive microenvironment significantly undermines the efficacy of immune checkpoint blockade and CAR-T cell therapies, representing a major reason for non-responsiveness in a subset of patients. Consequently, therapeutic strategies aimed at reprogramming TAMs toward pro-inflammatory and anti-tumor functional states have emerged as a promising direction in cancer immunotherapy (44, 45). Innovative approaches such as exosome-based therapies, nanoparticles, and CAR-macrophage therapies have demonstrated significant regulatory potential in preclinical studies (46–48).

Recent research in prostate cancer treatment highlights several key challenges and innovative strategies. First, although androgen deprivation therapy inhibits tumor growth, it paradoxically recruits pro-tumoral TAMs and dysfunctional T cells (49); targeted delivery of cGAMP to TAMs can activate the STING pathway, enhance anti-tumor immunity, and delay the progression of castration-resistant prostate cancer (CRPC) (50). Second, approximately 50% of metastatic CRPC cases develop therapeutic resistance due to aberrations in the PTEN/PI3K pathway; targeting lactate-mediated crosstalk between tumor cells and macrophages can remodel immunometabolic balance and improve the sustained efficacy of combination therapies (51, 52). Furthermore, to address the limitations of ferroptosis therapy, novel nanoparticle systems can simultaneously induce tumor cell death, activate dendritic cells, and pro-inflammatory macrophage-associated states, significantly enhancing innate immune responses and synergizing with immune checkpoint inhibitors (53, 54). Another strategy involves modulating macrophage activation states through UBC9 inhibition to reinforce a STAT4-driven pro-inflammatory phenotype, effectively reversing TAM-mediated immunosuppression and enhancing CD8+ T cell anti-tumor activity (55). Nanotechnology-based delivery platforms capable of remodeling the TME through immune cell modulation and enhanced antigen presentation offer a promising direction for combinatorial immunotherapy strategies (47). Collectively, these findings indicate that integrating immunometabolic regulation, targeted delivery, and combination treatment strategies represents a transformative approach for prostate cancer therapy, urgently awaiting clinical validation.

### 4.5 Clinical trials targeting TAMs in prostate cancer: challenges and opportunities

Reprogramming TAMs has emerged as a central therapeutic strategy to modulate the TME in PCa. Although extensive efforts have been made to target TAMs, clinical outcomes have been modest, underscoring the challenges of overcoming the immunologically cold nature of PCa. Initial approaches focused on inhibiting the CSF-1R pathway to reduce immunosuppressive, tumor-promoting TAM populations. A Phase I study of the CSF-1R monoclonal antibody LY3022855 in mCRPC patients demonstrated a reduction in pro-inflammatory monocytes and disease stabilization in a subset of patients, indicating potential immunomodulatory effects (56). However, subsequent trials evaluating other CSF-1R inhibitors, such as cabiralizumab and PLX3397, either alone or in combination with immune checkpoint inhibitors, failed to demonstrate significant clinical benefits, leading to the termination of several studies. Mechanistically, the limited activity of CSF1R inhibition as monotherapy may reflect the redundancy and adaptability of myeloid programs within the prostate cancer microenvironment. TAM depletion alone may be offset by alternative recruitment pathways, compensatory functional reprogramming of residual myeloid populations, or the persistence of other immunosuppressive components such as myeloid-derived suppressor cells and regulatory T cells. Importantly, these clinical observations provide a plausible translational explanation for our bibliometric finding that “Novel combination strategy” emerged as the largest co-citation cluster. In other words, the shift toward combination strategies in the atlas is not merely a publication trend, but likely reflects the field’s response to the biological insufficiency of single-pathway interventions in an immunologically cold and highly adaptive tumor microenvironment. This integrative perspective also helps explain why emerging hotspot themes such as lactate metabolism, ferroptosis-related pathways, and nanodelivery systems are increasingly relevant to translational research. Immunometabolic interventions may help disrupt tumor–myeloid crosstalk and reverse macrophage-associated immune suppression; ferroptosis-related approaches may enhance inflammatory signaling and antigen release; and nanodelivery platforms may improve the spatial precision and combinatorial delivery of macrophage-modulating agents, checkpoint inhibitors, or innate immune agonists. Together, these findings suggest that depletion-only strategies are unlikely to be sufficient and that the field is increasingly shifting toward rational combination regimens designed to reprogram TAM function while restoring effective anti-tumor T-cell activity, consistent with the “Novel combination strategy” cluster identified in our bibliometric analysis. Alternative strategies are currently under investigation. The leukocyte adhesion protein-1 analog GB1275, which targets CD11b/CD18 integrin, is being evaluated in a Phase 1/2 trial for treatment-resistant solid tumors, including mCRPC. Preclinical studies indicate that its mechanism involves suppressing the migration of myeloid-derived suppressor cells and TAMs, with potential synergy when combined with checkpoint inhibitors (57). Other innovative approaches include targeting the CD47-SIRPα axis to enhance macrophage phagocytosis. Given the limited efficacy of monotherapies, combination regimens have gained increasing attention. Multimodal strategies—such as combining androgen deprivation therapy with PI3K inhibitors and PD-1 blockade—are being evaluated in trials including IPATential-150 and CAPItello-281 for PTEN-deficient mCRPC, based on evidence that these combinations can enhance macrophage-mediated phagocytosis (58). In bone-metastatic CRPC, where macrophages are a dominant immune component, therapies combining ADT with inhibitors of the CCL2/CCR2 axis or CSF-1R are being explored to disrupt macrophage recruitment and function (59). Additionally, vaccine-based approaches such as GVAX, combined with CTLA-4 inhibition, aim to leverage the antigen-presenting functions of macrophages and dendritic cells (60). Despite these efforts, trials like KEYNOTE-199 have confirmed that PD-1 blockade alone yields low response rates in unselected mCRPC patients. Across the representative trials, unsuccessful outcomes commonly share several patterns: (i) TAM depletion or blockade strategies were often tested in heavily pretreated, immunologically “cold” mCRPC populations, where baseline T-cell infiltration and antigen presentation may be insufficient for downstream immune activation; (ii) endpoints were heterogeneous and frequently not designed to capture microenvironmental remodeling (e.g., on-treatment TAM signatures, spatial immune reorganization), limiting the ability to identify biologically responsive subgroups; and (iii) lack of biomarker-driven enrichment may have diluted potential signals of activity, as TAM-dominant phenotypes (e.g., CD163/CD206-high or SPP1+ macrophage-enriched tumors) were not consistently selected.

Based on these patterns and our bibliometric hotspots, future translational strategies may benefit from: (i) shifting from depletion-only approaches toward TAM reprogramming strategies that promote pro-inflammatory, antigen-presenting functions; (ii) rational combinations that align with the “novel combination strategy” cluster—such as CSF1R/CCR2-axis modulation plus immune checkpoint blockade, radiotherapy, vaccines, or STING-based approaches to enhance innate priming; (iii) incorporating immunometabolic interventions highlighted by emerging keywords (e.g., lactate-related signaling, ferroptosis-associated pathways) to disrupt tumor–myeloid crosstalk; and (iv) adopting biomarker-driven trial designs using baseline and on-treatment tissue profiling (single-cell and spatial transcriptomics) to identify actionable TAM subpopulations and to define pharmacodynamic endpoints. Future studies should prioritize biomarker-driven patient selection—using markers such as CD163 or CD206—and focus on repolarizing TAMs rather than solely depleting them. Integrating single-cell analyses and spatial transcriptomics may help identify actionable macrophage subpopulations and optimize therapeutic combinations.

In summary, while TAM-targeted therapy represents a promising approach in PCa, current clinical evidence suggests that depletion-only or single-agent strategies are unlikely to be sufficient. Instead, the field is increasingly moving toward biomarker-informed combination regimens that integrate TAM reprogramming with immune activation, metabolic intervention, or precision delivery systems—an evolution that closely mirrors the “Novel combination strategy” cluster identified in our bibliometric analysis.

### 4.6 Artificial intelligence and multi-omics integration for TAM-focused research in prostate cancer

Emerging evidence highlights the pivotal role of single-cell technologies, spatial transcriptomics, and integrative multi-omics approaches in refining our understanding of TAM heterogeneity and tumor–immune interactions in prostate cancer (61, 62). Recent prostate cancer-specific studies have moved beyond generalized descriptions of the tumor microenvironment and begun to define spatially and transcriptionally distinct myeloid ecosystems (63, 64). For example, single-cell profiling across the disease continuum showed that SPP1^high^ tumor-associated macrophages become enriched during progression to metastatic castration-resistant prostate cancer and are associated with immunotherapy resistance, with reduced responsiveness to CSF1R-targeted intervention (63). In parallel, integrated single-cell, spatial transcriptomic, and multiplex immunofluorescence analyses identified SPP1+/TREM2+ TAMs as a metastasis-enriched macrophage population associated with adverse clinical outcome, while blockade of SPP1 enhanced anti-PD-1 efficacy in preclinical prostate cancer models (64). Spatial and multi-omics studies have further shown that FAP+ fibroblasts and SPP1+ macrophages colocalize in prostate tumor tissues and may interact through pathways such as CSF1/CSF1R and CXCL/ACKR1, suggesting a stromal–myeloid axis of translational relevance (61). In addition, recent spatial transcriptomic work has highlighted the association between epithelial club-like cell states and immunosuppressive myeloid inflammation, providing a broader context for understanding how treatment resistance may be linked to specific immune niches in prostate cancer (65). Together, these studies suggest that future AI-assisted and multi-omics approaches should not only stratify patients computationally, but also focus on identifying actionable prostate cancer-specific TAM states, spatial immune niches, and stromal–myeloid interaction programs for biomarker-guided therapeutic development (63, 65).

### 4.7 Suggestions for future research

Future research on TAM-focused therapy in prostate cancer should move beyond isolated pathway inhibition and prioritize biomarker-guided combination strategies. In light of the bibliometric finding that “Novel combination strategy” represents the largest co-citation cluster, emerging hotspots such as lactate metabolism, ferroptosis-related pathways, and nanodelivery systems may be better understood not as separate directions, but as complementary modules for next-generation combinatorial design (66, 67). These approaches should be complemented by optimizing bioinspired nanodelivery systems such as reprogrammed microparticles and exosomes that target tumor-specific markers including FR-β and CD206 (68, 69). Such systems enable precise delivery of therapeutic agents including STING agonists or ferroptosis inducers, thereby synergistically promoting pro-inflammatory, anti-tumor macrophage reprogramming while enhancing innate immune responses and creating a more robust antitumor effect (50, 70). Concurrent efforts should focus on elucidating the combined mechanisms of immune checkpoint inhibitors such as αPD-1 with metabolic and ferroptosis therapies, particularly regarding how dynamic TAM-CD8+ T cell interactions amplify antitumor effects (71, 72). The integration of single-cell sequencing with clinical data will help identify TAM polarization markers such as STAT4 and PD-1, providing critical evidence for clinical trials targeting molecules like UBC9 (73–75). Furthermore, combining multi-omics with AI technologies to analyze TAM heterogeneity could enable dynamic monitoring of functional states and guide personalized treatment strategies (76). Ultimately, these approaches must integrate metabolic regulation, nanotechnology, and immune engineering. Systematic validation through preclinical models including genetically engineered mice and real-world data such as patient biopsies will be essential to overcome therapeutic barriers posed by the immunosuppressive microenvironment in prostate cancer, driving innovation from basic research to clinical translation (77, 78). Importantly, the bibliometric hotspots identified in this study suggest several promising directions for future research. For example, emerging keywords related to metabolic reprogramming indicate increasing attention to immunometabolic regulation of TAMs, including lactate metabolism and histone lactylation. In addition, ferroptosis-related pathways and macrophage-driven immunometabolic interactions may represent potential therapeutic targets. From a translational perspective, combination strategies integrating TAM modulation, immune checkpoint blockade, and nanotechnology-based drug delivery systems may offer new opportunities for improving immunotherapy efficacy in prostate cancer.

### 4.8 Limitations

First, the bibliometric data were retrieved from the WoSCC as the primary database for bibliometric analysis. Although WoSCC is widely recognized as a high-quality source with standardized citation metadata, the exclusion of other databases such as Scopus, PubMed, or CNKI may have resulted in incomplete literature coverage, particularly for emerging journals or non-English publications, and may therefore have introduced database selection bias. Second, the analysis was restricted to English-language articles and reviews, which may have excluded important studies published in other languages and thus introduced language bias. Third, no formal quality assessment of the included literature was conducted. As bibliometric analyses primarily rely on citation-based indicators, highly cited publications may not necessarily reflect methodological quality or clinical impact. Fourth, grey literature such as conference abstracts, dissertations, and non-indexed reports was not included in the dataset, which may limit the comprehensiveness of the analyzed research landscape. In addition, because bibliometric mapping is inherently dependent on author keywords, indexed terms, and citation patterns in the existing literature, it may preferentially capture historically dominant concepts such as the M1/M2 polarization framework while underrepresenting newly emerging TAM subpopulations and functionally defined macrophage states identified by single-cell and spatial transcriptomic approaches.

Future bibliometric studies could address these limitations by incorporating multiple databases, expanding language inclusion criteria, and applying additional quality assessment frameworks to stratify the included literature. In addition, integrating continuously updated clinical trial registries and real-world clinical datasets may further enhance the translational relevance of bibliometric findings.

## 5 Conclusion

This study provides a bibliometric analysis of global research trends in prostate cancer macrophage polarization from 2005 to 2024, analyzing 621 publications through cluster analysis, citation networks, and hotspot mapping. The findings reveal sustained growth in research output, with China and the United States as the most productive contributors, while Cancer Research emerged as the most cited journal. Despite this progress, international collaborations remain limited, highlighting the need for stronger academic exchange mechanisms. The analysis further identifies emerging research frontiers that are shaping the field’s future direction, offering both a synthesis of historical development and strategic guidance for future investigations.

## Data Availability

The original contributions presented in the study are included in the article/**Supplementary Material**. Further inquiries can be directed to the corresponding authors.

## Supplementary material

Supplementary Figure 1 **(A)** Collaboration network radar plot in prostate cancer macrophage polarization research. **(B)** Co-collaborating country network.

Supplementary Figure 2 Author analysis in prostate cancer macrophage polarization research.
