# Supplementary figures and images for: Mapping research trends in macrophage polarization and immunotherapeutic potential in prostate cancer: a bibliometric and visual analysis
Source: Front Oncol. 2026 May 4;16:1784198. doi: 10.3389/fonc.2026.1784198 (PMC13180588; doi:10.3389/fonc.2026.1784198)

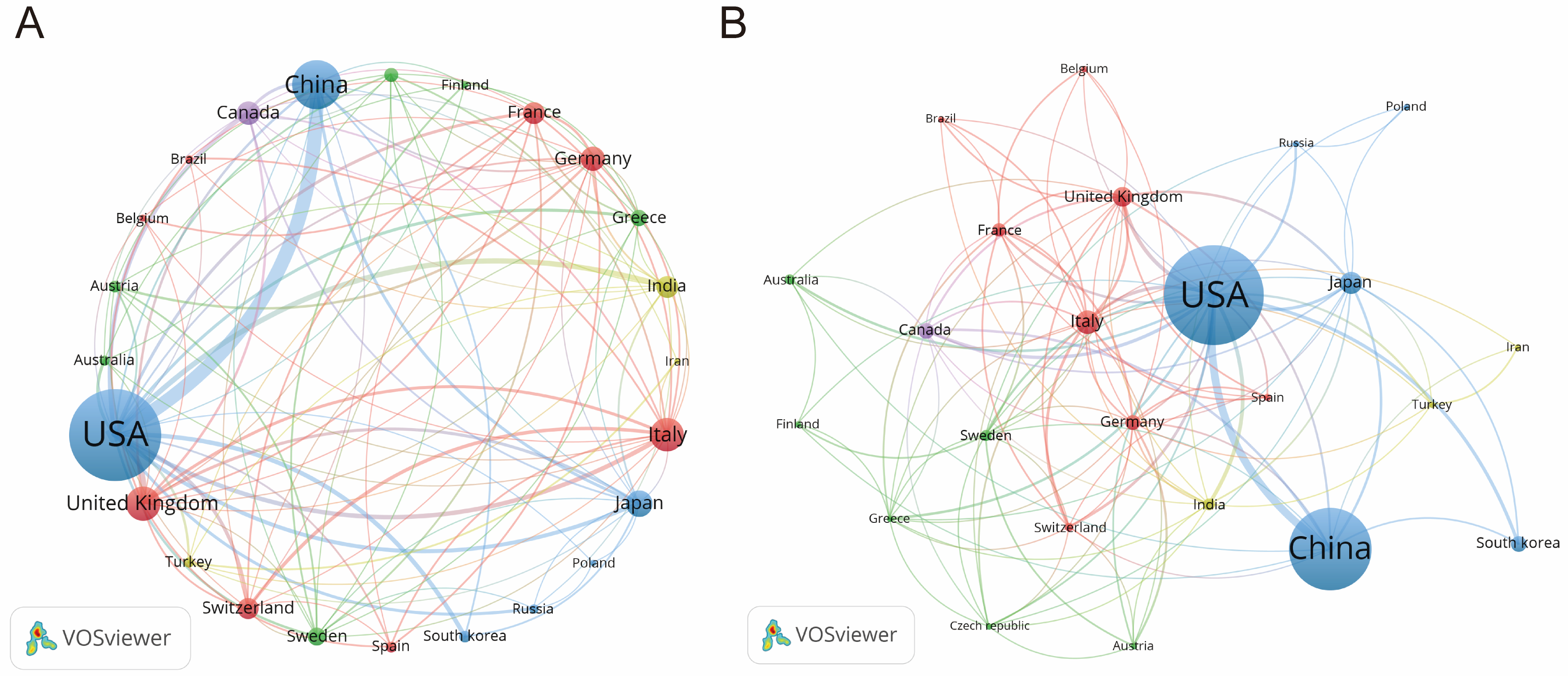

Supplement: Supplementary Figure 1 — (A) Collaboration network radar plot in prostate cancer macrophage polarization research. (B) Co-collaborating country network. [file Image1.tiff]

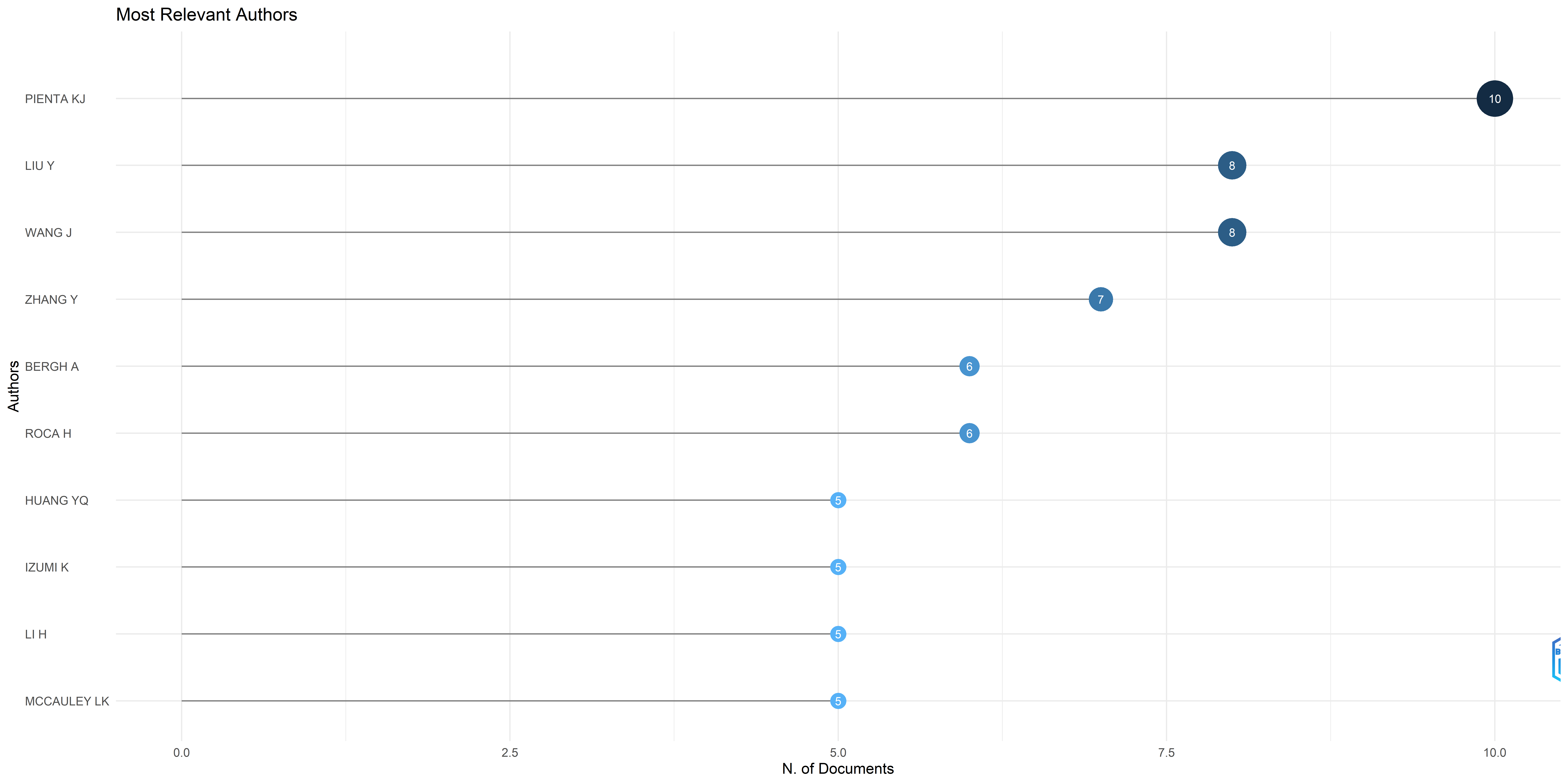

Supplement: Supplementary Figure 2 — Author analysis in prostate cancer macrophage polarization research. [file Image2.tiff]
